# Valley Fever (Coccidioidomycosis) Awareness — California, 2016–2017

**DOI:** 10.15585/mmwr.mm6942a2

**Published:** 2020-10-23

**Authors:** Glorietta Hurd-Kundeti, Gail L. Sondermeyer Cooksey, Seema Jain, Duc J. Vugia

**Affiliations:** 1 Infectious Diseases Branch, Division of Communicable Disease Control, California Department of Public Health.

Valley fever (coccidioidomycosis) is endemic in the southwestern United States and caused by inhalation of *Coccidioides* spp. fungal spores from soil or dust; 97% of U.S. Valley fever cases are reported from Arizona and California (*1*). In California, Valley fever incidence increased 213% from 2014 to 2018 (*2*). In 2016, the California Department of Public Health (CDPH) added three questions to the adult California Behavioral Risk Factor Surveillance System (BRFSS) survey to better understand whether Californians had heard of Valley fever, knew the environmental risk where they live, and knew who is at risk for severe disease. A total of 2,893 BRFSS respondents aged ≥18 years answered at least one Valley fever question. Using the weighted California population, 42.4% of respondents reported general awareness of Valley fever; awareness was lowest among adults aged 18–44 years (32.9%) and Hispanic persons (26.4%). In addition, despite higher percentages reporting awareness of Valley fever, only 25.0% of persons living in a high-incidence region and 3.0% of persons living in a moderate-incidence region were aware that they lived in areas where *Coccidioides* spp. exist. Among persons with one or more risk factors for severe disease, 50.8% reported having heard about Valley fever, but only 3.5% knew they were at increased risk for severe disease. The findings from this survey helped to inform a statewide Valley fever awareness campaign implemented during 2019–2020 and to guide outreach to persons living in high- and moderate-incidence regions in California and potentially other southwestern states or who are at risk for severe disease.

Valley fever usually is a self-limited illness with cough, fever, chest pain, or fatigue; however, some persons develop severe disease, and in rare cases, death occurs (*3*). Persons at risk for severe disease include adults aged ≥65 years, Black persons, Filipino persons, pregnant women, persons who smoke, and persons with diabetes or weakened immune systems (*3*–*5*). Because there is no vaccine and *Coccidioides* is an environmental pathogen, public awareness of Valley fever, particularly in high- and moderate-incidence regions and among groups at risk for severe disease, can aid in earlier disease recognition and management. In Arizona, analysis of enhanced Valley fever surveillance suggested that increasing public and provider education might reduce unnecessary treatment, relieve patient anxiety, and improve early recognition, diagnosis, and proper treatment (*6*). Given recent increases in Valley fever incidence in California, CDPH has aimed to increase educational efforts in an evidence-based manner, with an extended statewide campaign during 2019–2020. 

The California BRFSS is a telephone survey that collects data on health-related behaviors using random-digit dialing of landline and cell phone numbers (*7*), which afforded CDPH an opportunity to collect baseline information on whether Californians knew of Valley fever, risks for environmental transmission, or becoming severely ill. To assess Valley fever awareness, 3,485 California residents in the February 2016–February 2017 BRFSS survey were asked three Valley fever questions. Respondents were excluded from all analyses if sex, age, or race/ethnicity data were missing. Additional respondents were excluded from analyses of individual questions if they replied, “Don’t know,” refused to answer, or if data were otherwise missing. BRFSS survey design methodology and California BRFSS weighting were used to generate population response estimates (*7*).

First, general Valley fever awareness was assessed by asking, “Have you heard about the fungal disease called Valley fever, also known as coccidioidomycosis or cocci?” Second, environmental risk awareness was assessed by asking “The Valley fever fungus exists naturally in the soil in some areas, and persons living in these areas can get infected. Do you live in an area where the Valley fever fungus exists?” Third, knowledge of being at increased risk for severe disease was assessed by asking, “Some persons are at increased risk for severe Valley fever if infected. Are you one of these persons at increased risk for severe Valley fever?” The percentages of “Yes” responses for each question were analyzed by sex, age, race/ethnicity, severe disease risk groups, and incidence region as appropriate.

Groups at risk for severe disease were defined as adults aged ≥65 years, Black persons, Filipino persons, and persons with prediabetes or diabetes or who currently smoke. Incidence regions were defined based on the median county-specific number of Valley fever cases per 100,000 population per year during 2012–2017 and categorized into these regions: high incidence (≥10 per 100,000 population: Fresno, Kern, Kings, Madera, Merced, Monterey, San Joaquin, San Luis Obispo, Stanislaus, and Tulare counties), moderate incidence (2–9 cases per 100,000: Alameda, Calaveras, Contra Costa, Imperial, Los Angeles, Mariposa, Orange, Riverside, Santa Barbara, San Bernardino, Santa Cruz, San Diego, Solano, Tuolumne, and Ventura counties), and low incidence (<2 per 100,000: all other California counties). A first order Rao-Scott chi-squared test was used to compute p-values with <0.05 considered statistically significant. All analyses were conducted using SAS (version 9.4; SAS Institute).

Among 3,485 Californians surveyed, 2,893 (83.0%) responded to at least one Valley fever question, and 592 (17.0%) were excluded for not responding to any Valley fever question. After exclusion for missing data and weighting adjustment, 2,851 respondents were included in the analyses, with varying numbers of respondents for each question (range = 99.1%–99.6%).

Statewide, 42.4% of 2,824 respondents to the question, “Have you heard of Valley fever?” answered affirmatively, including 66.3%, 35.1%, and 45.0% in the high-, moderate-, and low-incidence regions, respectively (Table 1). Awareness was highest among adults aged ≥65 years (61.1%) and lowest among those aged 18–44 years (32.9%), and Hispanic persons (26.4%).

**TABLE 1:** Respondents* who had ever heard of Valley fever,^†^ by selected region and characteristics — Behavioral Risk Factor Surveillance System survey, California, 2016–2017

Characteristic	No.^§^	% who said yes	% of weighted state population who said yes (95% CI)	p-value
Statewide totals	2,824	44.0	42.4 (39.1–45.7)	—
**Sex**				
Female	1,501	45.7	41.1 (36.3–45.9)	0.427
Male	1,323	42.1	43.7 (39.3–48.2)	
**Age group (yrs)**				
18–44	1,049	29.4	32.9 (27.7–38.1)	<0.001
45–64	1,068	47.9	48.7 (43.8–53.7)	
≥65	707	59.8	61.1 (56.1–66.1)	
**Race/Ethnicity**				
White, NH	1,420	57.6	57.7 (53.7–61.7)	<0.001
Hispanic	925	25.6	26.4 (20.7–32.0)	
Non-White, NH	479	39.2	34.1 (26.2–42.1)	
**High-incidence region^¶^**				
**Region total**	**399**	**71.7**	**66.3 (53.5–79.1)**	**—**
**Sex**				
Female	237	70.9	61.7 (43.6–79.8)	0.238
Male	162	72.8	74.1 (61.6–86.5)	
**Age group (yrs)**				
18–44	149	59.1	53.5 (34.6–72.3)	<0.001
45–64	153	76.5	83.5 (76.2–90.8)	
≥65	97	83.5	85.3 (77.3–93.3)	
**Race/Ethnicity**				
White, NH	190	83.7	85.4 (77.8–93.0)	0.0021
Hispanic	170	60.6	58.6 (43.0–74.2)	
Non-White, NH	39	61.5	38.3 (1.5–75.1)	
Moderate-incidence region**				
**Region total**	**1,727**	**37.6**	**35.1 (31.1–39.1)**	**—**
**Sex**				
Female	919	39.1	33.4 (28.2–38.6)	0.387
Male	808	35.9	36.9 (30.9–42.8)	
**Age group (yrs)**				
18–44	654	23.7	25.8 (19.9–31.8)	<0.001
45–64	665	41.2	42.5 (36.2–48.9)	
≥65	408	53.9	55.1 (48.1–62.1)	
**Race/Ethnicity**				
White, NH	791	54.5	54.4 (48.7–60.1)	<0.001
Hispanic	626	17.1	17.0 (11.5–22.5)	
Non-White, NH	310	35.8	30.4 (21.5–39.4)	
**Low-incidence region^††^**				
**Region Total**	**576**	**44.1**	**45.0 (38.0–52.0)**	**—**
**Sex**				
Female	274	46.7	43.8 (33.1–54.5)	0.786
Male	302	41.7	45.7 (37.0–54.4)	
**Age group (yrs)**				
18–44	213	27.2	37.6 (25.6–49.6)	0.092
45–64	205	50.7	47.4 (36.3–58.6)	
≥65	158	58.2	56.6 (46.1–67.1)	
**Race/Ethnicity**				
White, NH	346	52.0	51.7 (43.6–59.7)	0.035
Hispanic	114	21.1	25.7 (11.8–39.7)	
Non-White, NH	116	43.1	43.0 (27.1–58.9)	

**Abbreviations:** CI = confidence interval; NH = non-Hispanic.

* Based on the weighted California percentage of respondents.

^†^ Based on response to question 1: “Have you heard about the fungal disease called Valley Fever, also known as coccidioidomycosis or cocci?”

^§^ Number represents adjusted survey counts, where responses missing either sex, age, or race and ethnicity values was removed from the analysis; respondents missing county information were removed from regional analysis.

^¶^ High-incidence region = ≥10 cases per 100,000 population (Fresno, Kern, Kings, Madera, Merced, Monterey, San Joaquin, San Luis Obispo, Stanislaus, and Tulare counties).

** Moderate-incidence region = 2–9 cases per 100,000 population (Alameda, Calaveras, Contra Costa, Imperial, Los Angeles, Mariposa, Orange, Riverside, Santa Barbara, San Bernardino, Santa Cruz, San Diego, Solano, Tuolumne, and Ventura counties).

**^†† ^**Low-incidence region = <2 cases per 100,000 population (all other California counties).

Statewide, 6.1% of 2,837 participants responded affirmatively to the question, “Do you live in an area where the Valley fever fungus exists?” including 25.0% in the high-incidence region and 3.0% each in the moderate- and low-incidence regions (Table 2). In the high-incidence region, environmental awareness was highest among adults aged ≥65 years (48.6%), and non-Hispanic White persons (40.7%); and lowest among adults aged 18–44 years (10.5%) and Hispanic persons (11.3%). In the moderate-incidence region, <5% of all demographic groups responded affirmatively to the environmental risk question.

**TABLE 2:** Percentage of Behavioral Risk Factor Surveillance System survey respondents* who indicated they live in an area where the Valley fever fungus exists, by selected region and characteristics — California, 2016–2017

Characteristic	No.^†^	% who said yes	% of weighted state population who said yes (95% CI)	p-value
**High-incidence region^§^**				
Total	401	33.2	25.0 (18.0–32.0)	—
**Sex**				
Female	238	31.9	22.2 (13.3–31.1)	0.332
Male	163	35.0	28.7 (18.2–39.2)	
**Age group (yrs)**				
18–44	149	16.8	10.5 (4.3–16.6)	<0.001
45–64	153	39.9	42.0 (30.0–54.1)	
≥65	99	47.5	48.6 (35.6–61.5)	
**Race/Ethnicity**				
White, NH	192	43.2	40.7 (30.6–50.7)	0.003
Hispanic	170	22.9	11.3 (6.1–16.4)	
Non-White, NH	39	28.2	16.5 (0.0–36.0)	
**Moderate-incidence region^¶^**				
Total	1,737	3.5	3.0 (2.0–4.0)	—
**Sex**				
Female	928	2.6	2.5 (1.0–4.1)	0.650
Male	809	4.4	3.0 (1.6–4.5)	
**Age group (yrs)**				
18–44	654	2.6	2.6 (0.9–4.3)	0.800
45–64	666	4.7	2.8 (1.5–4.0)	
≥65	417	2.9	3.6 (0.9–6.2)	
**Race/Ethnicity**				
White, NH	798	4.5	4.1 (2.0–6.3)	0.104
Hispanic	627	2.7	2.2 (0.9–3.5)	
Non-White, NH	312	2.2	1.5 (0.0–3.1)	
**Low-incidence region**				
Total	577	4.3	3.0 (1.2–4.0)	—
**Sex**				
Female	273	3.3	2.1 (0.2–3.9)	0.499
Male	304	5.3	3.0 (0.9–5.1)	
**Age group (yrs)**				
18–44	214	2.3	1.6 (0.0–3.4)	0.387
45–64	204	5.4	2.9 (0.4–5.5)	
≥65	159	5.7	4.2 (0.7–7.7)	
**Race/Ethnicity**				
White, NH	348	4.9	3.4 (1.1–5.7)	0.116
Hispanic	114	6.1	3.1 (0.1–6.1)	
Non-White, NH	115	0.9	0.5 (0.0–1.5)	

**Abbreviations:** CI = confidence interval; NH = non-Hispanic.

* Based on weighted California percentage of respondents.

^†^ Number represents adjusted survey counts, where responses missing either sex, age, or race and ethnicity values was removed from the analysis; respondents missing county information were removed from regional analysis.

^§^ High-incidence region = ≥10 cases per 100,000 population (Fresno, Kern, Kings, Madera, Merced, Monterey, San Joaquin, San Luis Obispo, Stanislaus, and Tulare counties).

^¶^ Moderate-incidence region = 2–9 cases per 100,000 population (Alameda, Calaveras, Contra Costa, Imperial, Los Angeles, Mariposa, Orange, Riverside, Santa Barbara, San Bernardino, Santa Cruz, San Diego, Solano, Tuolumne, and Ventura counties).

** Low-incidence region = <2 cases per 100,000 population (all other California counties).

Among 2,841 respondents to the severe Valley fever risk question, 1,272 (44.7%) had one or more risk factors for severe Valley fever based on BRFSS data (Table 3). Of those with one or more risk factors for severe Valley fever, 50.8% reported general Valley fever awareness, but only 3.5% responded that they were at increased risk for severe Valley Fever. When stratified by risk factors, which were not mutually exclusive, Black persons had both the lowest general Valley fever awareness (37.1%) and the lowest awareness of being at increased risk for severe disease (1.3%). Filipino persons and adults aged ≥65 years had higher general awareness of Valley fever (61.6% and 61.1%, respectively) but not for being at increased risk for severe disease (5.9% and 3.4%, respectively).

**TABLE 3:** Statewide respondents* with and without risk for severe Valley fever, by selected characteristics — California, 2016–2017

Risk factor	Survey question					
	“Have you heard about the fungal disease called Valley fever, also known as coccidioidomycosis or cocci?”			“Some individuals are at increased risk for severe Valley fever if infected. Are you one of these individuals at risk for severe Valley fever?”		
	No.^†^	% who said yes	Weighted California population % who said yes (95% CI)	No.*	% who said yes	Weighted California population % who said yes (95% CI)
**At risk for severe Valley fever**	**1,258**	**51.3**	**50.8 (45.5–56.1)**	**1,272**	**4.4**	**3.5 (2.1–5.0)**
Age ≥65 yrs	707	59.8	61.1 (56.1–66.1)	719	4.0	3.4 (1.9–4.9)
Diabetes and prediabetes	379	46.4	47.0 (38.1–55.8)	383	6.0	4.3 (1.9–6.6)
Current smoking	299	40.8	45.0 (32.9–57.1)	300	4.3	3.2 (0.6–5.8)
Black race	138	44.9	37.1 (26.6–47.7)	138	2.2	1.3 (0.0–2.8)
Filipino ethnicity	52	46.2	61.6 (38.1–85.0)	53	1.9	5.9 (0.0–17.1)
**Not at risk for severe Valley fever**	**1,566**	**38.1**	**37.1 (33.1–41.1)**	**1,569**	**2.8**	**2.0 (1.0–2.9)**

**Abbreviation:** CI = confidence interval.

* Based on weighted California percentage of respondents.

^†^ Number represents adjusted survey counts, for which responses missing either sex, age, or race and ethnicity values (i.e., Filipinos and Black persons) were removed from the analysis; specific risk groups were not mutually exclusive.

## Discussion

The 2016–2017 California BRFSS survey indicated that fewer than half of Californians had general Valley fever awareness, and awareness was lowest among persons living in moderate-incidence regions, adults aged <45 years, and non-White residents. In the high-incidence region, general Valley fever awareness (66.3%) was much higher than that in the moderate- and low-incidence region; suggesting that local Valley fever awareness efforts in the high-incidence region (e.g., by providers, public health, media, and support groups in Kern and neighboring counties) (*8*,*9*), have produced increased awareness. Despite that, only 25% persons living in the high-incidence region, and even fewer Hispanics (11.3%) and adults aged 18–44 years (10.5%) in this region, knew that *Coccidioides* spp. existed in this area. In the moderate-incidence region, which included southern California, accounting for >50% of the state’s population, environmental risk awareness was even lower (<5%) among all groups.

Among persons at increased risk for severe disease, approximately half had general Valley fever awareness but only 3.5% knew of their increased risk for severe disease. Raising Valley fever awareness in these populations at risk for severe disease is critical to increasing knowledge that could help reduce exposure to dust in areas where *Coccidioides* spp. exists; in addition, if persons become infected, recognizing their illness as Valley fever and seeking earlier clinical care might lead to improved outcomes.

The findings in this report are subject to at least four limitations. First, the number of respondents was relatively small; therefore, results might not be generalizable to the entire state population. Second, analyses were based on the respondent’s county of residence, which might differ from where *Coccidioides* spp. exposures might occur. Third, certain risk factors for severe disease could not be included because they were not available or prevalent in BRFSS, notably pregnancy and immunosuppression (e.g., treatment for cancer or human immunodeficiency virus infection). Finally, BRFSS weighting factors are based on the total California population and might not represent smaller geographic areas (*7*).

California’s population is projected to increase, particularly in areas where Valley fever incidence is high or increasing (*10*). Findings in this report indicated a need to raise Valley fever awareness statewide and helped guide the California Valley fever awareness campaign with outreach to persons living in high- and moderate-incidence regions and to persons at risk for severe disease.

**Summary**

**What is already known about this topic?**

Valley fever (coccidioidomycosis) incidence in California has increased significantly since 2014.

**What is added by this report?**

During 2016–2017, 42.4% of California Behavioral Risk Factor Surveillance System survey respondents reported general Valley fever awareness, but only 25.0% of persons living in a high-incidence region were aware that they lived where *Coccidioides* spp. exist. Among persons at increased risk for severe disease, only 3.5% knew that they were at increased risk.

**What are the implications for public health practice?**

Public awareness of Valley fever, particularly in high and moderate-incidence regions and among groups at risk for severe disease, can aid in earlier disease recognition and management. These survey results helped guide a statewide Valley fever awareness campaign in California and potentially might inform programs in other southwestern states where persons are at risk for severe disease.
