# *Erratum:* Vol. 69, No. 42

**DOI:** 10.15585/mmwr.mm6943a7

**Published:** 2020-10-30

In the report “Valley Fever (Coccidioidomycosis) Awareness — California, 2016–2017,” on page 1515, the e-mail address for the Corresponding author, Duc J. Vugia, was incorrect. The correct address is **duc.vugia@cdph.ca.gov**.

